# Development and Validation of a Radiomic-Based Model for Prediction of Intrahepatic Cholangiocarcinoma in Patients With Intrahepatic Lithiasis Complicated by Imagologically Diagnosed Mass

**DOI:** 10.3389/fonc.2020.598253

**Published:** 2021-01-07

**Authors:** Beihui Xue, Sunjie Wu, Minghua Zheng, Huanchang Jiang, Jun Chen, Zhenghao Jiang, Tian Tian, Yifan Tu, Huanhu Zhao, Xian Shen, Kuvaneshan Ramen, Xiuling Wu, Qiyu Zhang, Qiqiang Zeng, Xiangwu Zheng

**Affiliations:** ^1^ Radiological Department, The First Affiliated Hospital of Wenzhou Medical University, Wenzhou, China; ^2^ Department of Hepatology, The First Affiliated Hospital of Wenzhou Medical University, Wenzhou, China; ^3^ The First Clinical Medical College of Wenzhou Medical University, Wenzhou, China; ^4^ School of Pharmacy, Minzu University of China, Beijing, China; ^5^ The Second Affiliated Hospital and Yuying Children's Hospital of Wenzhou Medical University, Wenzhou, China; ^6^ Dr A. G Jeetoo Hospital, Port Louis, Mauritius; ^7^ Department of Pathology, The First Affiliated Hospital of Wenzhou Medical University, Wenzhou, China; ^8^ Department of Hepatobiliary Surgery, The First Affiliated Hospital of Wenzhou Medical University, Wenzhou, China

**Keywords:** intrahepatic cholangiocarcinoma, intrahepatic lithiasis, radiomics, risk factors, nomogram

## Abstract

**Background:**

This study was conducted with the intent to develop and validate a radiomic model capable of predicting intrahepatic cholangiocarcinoma (ICC) in patients with intrahepatic lithiasis (IHL) complicated by imagologically diagnosed mass (IM).

**Methods:**

A radiomic model was developed in a training cohort of 96 patients with IHL-IM from January 2005 to July 2019. Radiomic characteristics were obtained from arterial-phase computed tomography (CT) scans. The radiomic score (rad-score), based on radiomic features, was built by logistic regression after using the least absolute shrinkage and selection operator (LASSO) method. The rad-score and other independent predictors were incorporated into a novel comprehensive model. The performance of the Model was determined by its discrimination, calibration, and clinical usefulness. This model was externally validated in 35 consecutive patients.

**Results:**

The rad-score was able to discriminate ICC from IHL in both the training group (AUC 0.829, sensitivity 0.868, specificity 0.635, and accuracy 0.723) and the validation group (AUC 0.879, sensitivity 0.824, specificity 0.778, and accuracy 0.800). Furthermore, the comprehensive model that combined rad-score and clinical features was great in predicting IHL-ICC (AUC 0.902, sensitivity 0.771, specificity 0.923, and accuracy 0.862).

**Conclusions:**

The radiomic-based model holds promise as a novel and accurate tool for predicting IHL-ICC, which can identify lesions in IHL timely for hepatectomy or avoid unnecessary surgical resection.

## Introduction

Intrahepatic cholangiocarcinoma (ICC) is the second most prevalent liver malignancy following hepatocellular carcinoma, and its global disease incidence is increasing (1, 2). The risk factors for ICC are complex, but recently intrahepatic lithiasis (IHL) has been confirmed as a strong risk factor. High Odds ratios (ORs) have been found for developing ICC due to hepatolithiasis, up to 50 in Korea (3), six in China (4), and seven in Italy (5). Studies have reported that about 2.3 to 13.0% of patients with hepatolithiasis end up developing cholangiocarcinoma (6–11), and 65–70% of patients in Taiwan who underwent resection for cholangiocarcinoma suffer from concomitant hepatolithiasis (12, 13).

It is very difficult for a clinical surgeon to identify ICC early in patients with IHL because there are no specific symptoms and radiological features. Although tissue biopsy can be used to confirm a histological diagnosis, it is not routinely recommended in ICC (14), especially in IHL-ICC where ‘negative’ biopsy results do not exclude ICC given the significant potential for sampling error. The preoperative diagnosis for IHL-ICC is mainly obtained from a combination of imaging, serum carcinoembryonic antigen (CEA), and cancer antigen 19-9 (CA 19-9). However, the current diagnostic accuracy of IHL-ICC is low, generally ranging from 30 to 65% (7, 10, 11, 15, 16). Recently, we have increased the diagnostic accuracy to 78.5% through developing a nomogram for patients with IHL complicated by imagologically diagnosed mass (17). Despite this improvement, the accuracy of preoperative imaging diagnosis in the nomogram was still low because it was performed by two radiologists based on their experience. In recent years, radiomics has been introduced in clinic to identify liver tumors (18); however, no studies have used the radiomic approach for diagnosing IHL-ICC. Therefore, there is an urgent need to develop a radiomic model capable of improving the diagnostic accuracy of IHL-ICC.

In this study, we aimed to identify the radiomic features of IHL-ICC, develop a predictive model that combined radiomic score (rad-score) and clinical features for preoperative identification of ICC among patients with IHL, and also to validate its predictive capacity in an independent data sets.

## Patients and Methods

### Patients Selection

All patients involved in this retrospective study that constituted the training cohort were diagnosed with intrahepatic lithiasis (IHL) complicated by imagologically diagnosed mass (IM) (IHL-IM) and underwent hepatectomy at The First Affiliated Hospital of Wenzhou Medical University (WMU) from January 2005 to July 2019. The database from our hospital was screened meticulously to select the potentially eligible patients who were; (1) with pathological diagnosis of ICC or IHL and (2) with available high-quality contrast-enhanced computed tomography (CT) before surgical resection. The clinical characteristics of these qualified patients were recorded. This retrospective study was reviewed and approved by the Institutional Review Board (IRB) of the First Affiliated Hospital of WMU, and a waiver of written informed consent was granted by the IRB due to the retrospective nature of this study in which de-identified data were used and analyzed.

The patients for the external validation cohort were selected from the Second Affiliated Hospital of WMU, whose IRB approved the validation study.

**Figure 1:**
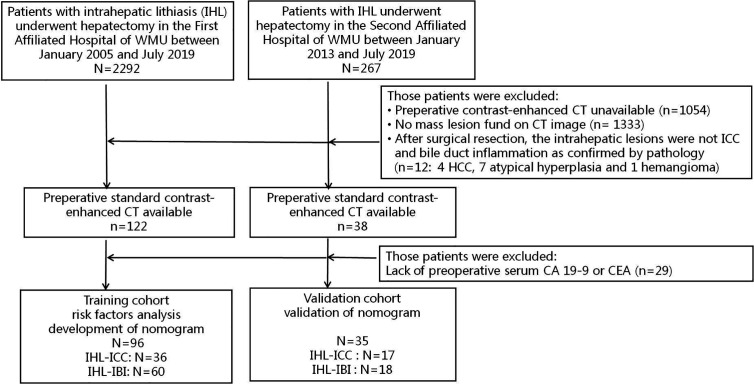
Proceeding flow of enrollment.

Details for the recruitment and selection criteria of the patients included in this study were shown in **Figure 1**.

### CT Image Acquirement, Tumor Segmentation, and Radiomic Feature Extraction

All patients were assessed with contrast-enhanced CT using the LifeX software tools (19). Two radiologists (BX and SW) who were blinded to the pathologic details, reviewed transverse CT images to determine respectively the features of the mass location and boundary.

**Figure 2:**
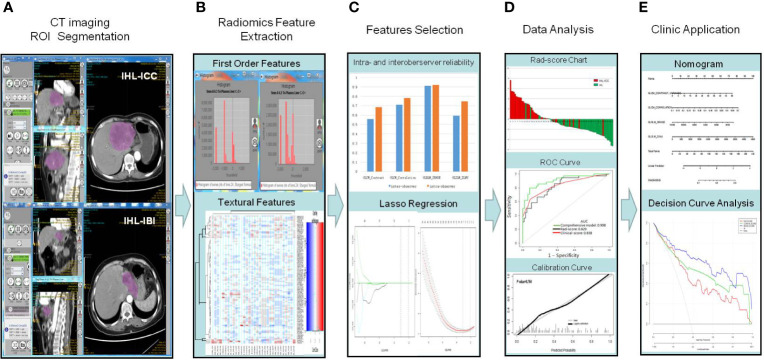
Workflow of required steps in this current study. **(A)** Manual segmentation on arterial phase CT scans; **(B)** Quantification of tumor intensity, shape, and texture through radiomic features collected by LIFEx software from inside the defined tumor contours on CT images. **(C)** For feature selection, two successive steps are the reliability assessment regarding the extracted features, followed by the LASSO method. A radiomic signature was obtained by combining the selected features by their respective coefficients, linearly. **(D)** By measuring the area under a receiver operating characteristic (ROC) curve and the calibration curve, the performance of the prediction model can be analyzed. **(E)** A radiomic nomogram was built in order to provide clinicians with a visual tool through the use of the selected covariates, followed by decision curve.

The radiomic workflow is depicted in **Figure 2**. Image feature extraction was performed on retrieved arterial phase CT images (5 mm thickness). The pre-processing procedure [*i.e.*, the uniform of window width (200 Hu), window level (45 Hu), and pixel size (512 × 512)] was undertaken before feature extraction. Manual segmentation of tumor regions of interest (ROI) was carried out by two different radiologists (BX and SW). Each transverse slice consisted of cuts made along the primary tumor contour. A total of fifty-two quantified texture features were extracted, including features from histogram-based matrix and shape-based matrix from the first order and features from gray-level co-occurrence matrix (GLCM), gray-level zone length matrix (GLZLM), neighborhood gray-level dependence matrix (NGLDM), and gray-level run length matrix (GLRLM) from second or higher order (20). A detailed description of all these characteristics can be found in https://www.lifexsoft.org/index.php/resources/19-texture/radiomic-features. All original data about extracted features are displayed in the **Supplementary Material 1** and **Supplementary Material 2**.

### Radiomic Feature Selection and Signature Construction

We devised a two-step procedure for dimensionality reduction and selection of robust features. Firstly, we calculated the intraobserver and interobserver reliability for each ROI based radiomic feature, extracted from 50 randomly chosen images. To assess interobserver reliability, the ROI segmentation was performed by two experts [one radiologist (reader 1, BX) and one hepatobiliary surgeon (reader 2, QZ)] who were blinded to both the clinical and pathologic details. To evaluate intraobserver reliability, reader 1 repeated the ROI segmentation and feature extraction procedure twice over a period of one month. The reliability was calculated by using intraclass correlation coefficient. Radiomic features with both intraobserver and interobserver intraclass correlation coefficient values greater than 0.55 (demonstrating at least moderate stability) were selected for subsequent investigation. Secondly, the least absolute shrinkage and selection operator (LASSO) logistic regression algorithm was applied to the training cohort in order to determine which ICC-related features had non-zero coefficients while being cross-validated 10 times by the penalty parameter. A radiomic signature was generated *via* a linear combination of selected features weighted by their respective coefficients (21).

### Development, Performance, and Validation of a Radiomic Nomogram

A radiomic model incorporating the radiomic signature, as well as independent risk factors that were obtained in our previous research for IHL-ICC (17), was constructed based on the results of the multivariate logistic regression analysis performed on the training cohort. A radiomic nomogram was then constructed in order to provide clinicians with a visual tool through the use of the selected covariates. Furthermore, a clinical model based on multivariate logistic regression analysis of candidate predictors, with the exception of radiomic signature, was developed. We calculated the area under the curve (AUC) of the receiver operating characteristic curve (ROC) to measure the discrimination performance of established models, and through the use of the DeLong algorithm (22), we compared the differences in AUC estimates between the various models. Calibration curves were graphed, through bootstrapping (resampled 1,000 times), to evaluate the predictive accuracy of the radiomic nomogram, followed by a Hosmer–Lemeshow test (P > 0.05 indicating good fit) (23). The performance of the radiomic model was then externally tested through an independent validation cohort.

### Clinical Utility of the Radiomic Nomogram

The net benefits at different threshold probabilities were quantified by a decision curve analysis (DCA) (24), thereby estimating the clinical utility of the established models in the validation cohort.

### Statistical Analysis

Numerical variables were compared by means of the *t*-test or Mann–Whitney U test, and categorical variables were compared using the χ2 test or Fisher’s exact test, where appropriate. Univariate and multivariate Cox regression analyses were performed to determine predictors of IHL-ICC. All variables with a *p-*value <0.05 in univariate analysis were selected for multivariate analysis. Statistical analyses were performed with the R software (version 3.4.4, http://www.R-project.org), the EmpowerStats software (www.empowerstats.com, X&Y solutions, Inc. Boston MA). The R package “glmnet” was used to perform LASSO binary logistic regression analysis; the “rms” package, to create the nomogram; and the “pROC” package, to analyze ROC curves. A two-sided *p*-value <0.05 was considered statistically significant.

## Results

### Demographic and Clinicopathological Characteristics

A total of 96 eligible patients were selected from the training cohort. Thirty-six of them were diagnosed with IHL-ICC, and 60 patients were diagnosed as IHL with intrahepatic biliary inflammation (IHL-IBI). Furthermore, 35 patients (17 IHL-ICC and 18 IHL-IBI) were included for validation. The detailed characteristics of the patients were summarized in **Table 1**. There were no significant differences regarding clinical and radiologic characteristics, in both the training and validation cohorts.

**Table 1:** Demographic and clinical characteristics of the study population.

	Training cohort			Validation cohort		
	IHL-IBI	IHL-ICC	*P* value	IHL-IBI	IHL-ICC	*P* value
Demographic or Characteristic	(n = 60)	(n = 36)		(n = 18)	(n = 17)	
Age, mean (SD)			0.01			0.73
<60 y	29 (48.33%)	8 (22.22%)		8 (44.44%)	6 (35.29%)	
≥60 y	31 (51.67%)	28 (77.78%)		10 (55.56%)	11 (64.71%)	
Sex (F/M)	37/23	22/14	0.96	11/7	10/6	1
Smoking	7 (11.67%)	6 (11.67%)	1	2 (11.11%)	2 (11.76%)	1
Alcohol	8 (13.33%)	9 (25%)	0.15	3 (16.67%)	4 (23.53%)	1
Personal cancer history	2 (3.33%)	2 (5.56%)	1	1 (5.56%)	0	1
Family cancer history	1 (1.67%)	0 (0%)	1	1 (5.56%)	1 (5.88%)	1
Inflammatory attacks within half a year (≥2 times)	12 (20.00%)	9 (25.00%)	0.06	4 (22.22%)	4 (23.53%)	1
Lesion size (cm), mean (SD)	5.42 (1.88)	5.79 (1.63)	0.48	5.27 (2.45)	5.98 (1.66)	0.35
Location of hepatolithiasis						
Left lobe	40 (66.67%)	26 (72.22%)	0.57	11 (61.11%)	13 (76.47%)	0.47
Right lobe	15 (25.00%)	8 (22.22%)	0.76	4 (22.22%)	3 (17.65%)	1
Left and right lobes	3 (5.00%)	2 (5.56%)	1	2 (11.11%)	1 (5.88%)	1
Lobus caudatus	2(3.33%)	0	1	1(5.56%)	0	1
Symptoms						
Abdominal pain	53 (88.30%)	30 (83.33%)	0.7	14 (77.78%)	12 (70.59%)	0.71
Fever	31 (51.67%)	12 (33.3%)	0.08	4 (22.22%)	3 (17.65%)	1
Vomiting	20 (33.30%)	6 (16.67%)	0.08	6 (33.33%)	2 (11.76%)	0.22
Jaundice	8 (13.30%)	3 (8.30%)	0.68	2 (11.11%)	2 (11.76%)	1
Weight loss	1 (1.67%)	1 (2.78%)	0.61	0	1 (5.88%)	1
Laboratory						
ALK (U/L), mean (SD)	218.70 (224.42)	226.94 (183.96)	0.17	166.52 (263.94)	192.84 (218.13)	0.38
γ-GT (U/L), mean (SD)	218.80 (261.40)	221.56 (188.88)	0.31	269.71 (332.59)	177.41 (291.32)	0.30
ALT (U/L), mean (SD)	75.75 (80.52)	55.89 (76.81)	0.11	83.22 (141.27)	47.77 (52.48)	0.28
Albumin (g/dl), mean (SD)	35.37(5.65)	35.54 (4.63)	0.9	38.19 (4.26)	36.66 (5.33)	0.55
PT (second), mean (SD)	14.18 (1.65)	15.50 (10.53)	0.42	14.84 (1.37)	13.49 (0.77)	0.62
CA 19-9 (U/ml), median (IQR)	42.35 (11.18, 407.42)	902.8 (28.6, 2020.80)	<0.01	16.26 (7.08, 130.80)	92.96 (4.2, 1200)	<0.01
CEA (μg/L), median (IQR)	1.80 (1.20, 2.30)	5.50 (2.10, 35.10)	<0.01	2.21 (1.2, 3.11)	5.15 (2.13, 31.88)	<0.01
AFP (μg/L), median (IQR)	2.48 (1.70, 3.66)	3.25 (2.10, 4.21)	0.07	2.64 (1.68, 3.66)	2.55 (1.78, 3.42)	0.87
CA 125 (U/ml), mean (SD)	12.37 (0.99)	1399.36 (3440.36)	0.09	28.58 (139.99)	122.71 (165.13)	0.01
Complication						
HBsAg+	4 (6.67%)	4 (11.11%)	0.65	2 (11.11%)	1 (5.88%)	1
HBcAb+	16 (26.67%)	8 (22.22%)	0.84	10 (55.56%)	10 (58.82%)	1
Diabetes	7 (11.67%)	2 (5.56%)	0.53	1 (5.56%)	0	1
Clinical Score, mean (SD)	−1.31 (1.05)	0.94 (1.97)	<0.01	−1.34 (1.02)	0.87 (1.97)	<0.01
Radiomic Score, mean (SD)	−1.32 (1.23)	0.54 (1.62)	<0.01	−0.86 (1.18)	0.55 (1.82)	<0.01

### Feature Selection and Radiomic Signature Construction

**Figure 3:**
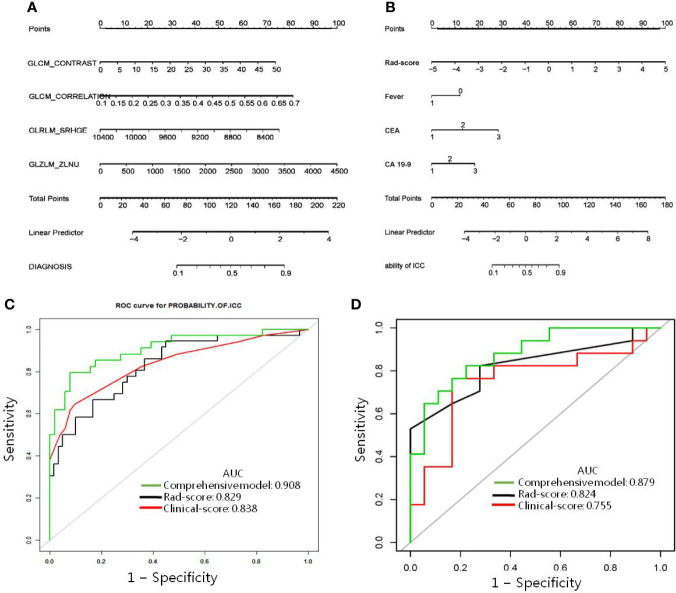
Radiomic nomogram designed with receiver operating characteristic curves. **(A)** The radiomic nomogram and **(B)** the comprehensive model was developed in the training cohort for predicting IHL-ICC. Comparison of ROC among the radiomic nomogram, clinical model, and comprehensive model for the prediction of IHL-ICC in the **(C)** training and **(D)** validation cohorts.

Of 52 extracted radiomic features, four ICC-related features with non-zero coefficients in the LASSO logistic regression model were obtained from the training cohort. The radiomic score used to calculate the novel radiomic signature was obtained by means of the following formula: rad-score = 9.79113 + 0.06519 *GLCM_CONTRAST_VARIANCE + 5.97425*GLCM_CORRELATION-0.00151*GLRLM_SRHGE + 0.00098*GLZLM_ZLNU (**Figure 3A**).

### Diagnostic Validation of Radiomic Signature and Clinical Prediction Models

The radiomic signature model exhibited promising discriminative ability for IHL-ICC and IHL-IBI in the training cohort. The AUC of the radiomic signature model was 0.829 [95% confidence interval (CI): 0.744, 0.910] with sensitivity 0.868, specificity 0.635, and accuracy 0.723 in training cohort (**Figure 3C**). Furthermore, by combining three independent factors (fever, CEA, and CA 19-9) in the training cohort, a clinical prediction model was constructed. The AUC of the nomogram for the clinical prediction model was 0.838 (95% CI, 0.747–0.928), with a sensitivity, specificity, and accuracy of 0.902, 0.647, 0.800 respectively (**Figure 3C**).

In the validation cohort, AUC of the radiomic signature model was 0.824 (95% CI: 0.768, 0.989) with sensitivity 0.824, specificity 0.778, and accuracy 0.800. The AUC of the nomogram for the clinical prediction model was 0.824 (95% CI, 0.681–0.966), with a sensitivity, specificity, and accuracy of 0.824, 0.722, 0.771 respectively (**Figure 3D**).

### Development, Performance, and Validation of Prediction Models

A comprehensive model incorporating two kinds of independent predictors (radiomic signature and clinical features) was developed, by using the following formula: comprehensive model = −0.87516 + 0.84946*rad-score −1.02770*1 (if with fever) + 1.11976*2 (if 5μg/L≥CEA≥3.75μg/L) + 2.41799*(if CEA > 5μg/L) + 0.64579*(if 143.15 U/ml≥CA 19-9≥37 U/ml) + 1.56721*(if CA 19-9>143.15 U/ml), and presented as a nomogram (**Figure 3B**). The model is capable of indicating a good fit, as proved the Hosmer–Lemeshow test (*p* = 0.764), and the calibration of the nomogram was likewise well-calibrated, as illustrated in **Figure 2D**. In the training cohort, the comprehensive model displayed the highest discrimination between IHL-ICC and IHL-IBI with an AUC of 0.908 (95% CI: 0.833, 0.970) (sensitivity 0.771, specificity 0.923, and accuracy 0.862); the detected AUC value was higher than that of the radiomic signature model (AUC, 0.829; *p* < 0.05) and clinical prediction model and (AUC, 0.838; *p* < 0.05) (**Figure 3C**). In the validation cohort, the comprehensive model presented the greatest AUC (0.879; 95% CI: 0.768, 0.990) as well, which confirms that the comprehensive model is capable of better predictive efficacy than either the radiomic signature model (AUC, 0.824; *p* < 0.05) or clinical prediction model alone (AUC, 0.755; *p* < 0.05) (**Figure 3D**).

### Clinical Use

**Figure 4:**
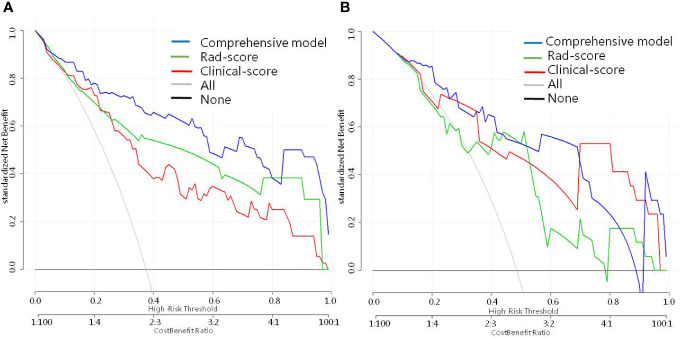
Decision curve analysis for every model in **(A)** the training and **(B)** validation dataset. The net benefit is measured by the y-axis, which is calculated by summing the benefits (true-positive findings) and deducting the harms (false-positive findings), while weighting the harms associated to the relative damage of undetected IHL-ICC when compared with the damage of being mistakenly diagnosed with HL-ICC.

The DCA for the radiomic nomogram, the clinical prediction model, and the comprehensive model are presented in **Figure 4**. The comprehensive model is capable of providing a better net benefit when predicting ICC in IHL patients, when compared with the other two models (demonstrated by the threshold probabilities of more than 10%), and particularly, in situations where there is no alternative prediction model available.

## Discussion

The accurate diagnosis for IHL patients with ICC is extremely important because it can facilitate the decision making with regard to surgical treatment at an early stage. The present work is the first attempt to propose a comprehensive model combined with radiomic and clinical signatures that can improve the current diagnostic accuracy standard of ICC in patients with IHL. The prediction model was validated internally and externally.

In a recent study, we had developed a nomogram to predict ICC for patients with IHL complicated by the presence of a imagologically diagnosed mass (17). However, the imagological diagnosis in the nomogram was made by radiologists. Even for experienced radiologists, the accuracy of diagnosis is still lower than 70% (10, 11, 15, 17). Detection of ICC in IHL is mainly dependent on imaging modalities because there are no specific symptoms in cases of IHL-ICC other than the clinical manifestation of hepatolithiasis.

ICC can be according to three types of morphological characteristics: mass-forming, periductal infiltrating, and intraductal growth. Of these three, mass-forming is the most common type and on CT scan, usually resembles a homogeneous low-attenuation mass with irregular peripheral enhancement, often accompanied by capsular retraction, peripheral intrahepatic duct dilation, and satellite nodules. If the dysplastic bile duct presents growth without mass formation, then it possesses the characteristics of a periductal infiltrating cholangiocarcinoma. Diffuse periductal thickening and increased enhancement can be observed in a dilated or irregularly narrowed intrahepatic duct. For patients with IHL, inflammatory pseudo-tumors or liver abscesses often occur at the site of intrahepatic stones, thus making it difficult to distinguish from mass-forming ICC, whereas proliferative cholangitis or inflammatory stenosis are difficult to distinguish from periductal infiltrating ICC. Furthermore, after long-term chronic inflammation, liver segments often become scarred and undergo fibrotic change (11), making IHL even more difficult to distinguish from ICC on imaging.

The radiomic technique can process high-throughput extraction of quantitative features that result in the conversion of images into mineable data and the subsequent analysis of these data for decision support, which draws a contrast with the traditional treatment of medical images as simple tools of visual interpretation. Radiomic data contain first-, second-, and higher-order statistics. The radiomic technique is very useful for IHL-ICC, which is highly heterogeneous and short of traditional imaging features. We used the LIFEx (A Freeware for Radiomic Feature Calculation) to implement these functions of ROI segmentation and radiomic feature extraction in a one-stop manner. The LASSO logistic regression algorithm can effectively solve the problem of multicollinearity among numerous extracted features and find meaningful feature parameters for a well constructed prediction model. In the present research, we got the higher-order radiomic features of IHL-ICC including GLCM_CONTRAST, GLCM_CORRELATION, GLRLM_SRHGE, and GLZLM_ZLNU that were obviously different from IHL-IBI. Finally, the radiomic model has improved the diagnostic accuracy for IHL-ICC to 0.72 which is higher than in our previous research (17) and others (10, 11, 15).

Furthermore, a comprehensive model incorporating two kinds of independent predictors (radiomic signature and clinical features) was developed for further improving the diagnostic accuracy for IHL-ICC. Based on our previous research, the clinical risk factor for IHL-ICC included biliary tract surgical history, fever, ascites, CA 19-9, and CEA. Here, we removed indicators that need to be judged subjectively, such as vomiting, and retained objective indicators including fever, CA 19-9, and CEA. The comprehensive model further improves the diagnostic accuracy to 86%, which is simpler and more convenient. As a non-invasive method, the comprehensive model for IHL-ICC would be a convenient application for clinicians.

There are several limitations to the present study. First, due to retrospective design and small sample, the potential selection bias cannot be excluded, which limits the accuracy and reliability of results. Second, when highlighting the outline of ROI areas, the variation between observed images should be deliberated. The inclusion of a computer-aided software, as used in this study, may help to reduce variation to some degree. Third, the texture features mined in this study were based solely on arterial phase CT images. Further investigation is needed to evaluate the performance of using either portal venous- or delayed-phase imaging or in combination, for predicting the malignant potential of IHL-IM. Furthermore, there are many different types of texture features and imaging processing software, so unifying the texture analysis would undoubtedly add rigor to the results obtained while spreading the application of this technology. Therefore, more investigation attempts are necessary for better estimation, especially large-scale prospective, and multicenter studies.

## Conclusions

A prediction nomogram based on CT radiomics was created and validated in this study. It was suitably utilized in order to simplify the individualized prediction of malignancy in IHL-IM patients. The radiomic-based model holds promise as a novel and accurate tool for predicting IHL-ICC, which can identify lesions in IHL, in a timely fashion, determining if there is a need for hepatectomy, avoiding unnecessary surgical resection.

## Data Availability Statement

The original contributions presented in the study are included in the article/**Supplementary Materials**. Further inquiries can be directed to the corresponding author.

## Ethics Statement

The studies involving human participants were reviewed and approved by the Institutional Review Board (IRB) of the First Affiliated Hospital of WMU. The patients/participants provided their written informed consent to participate in this study.

## Author Contributions

All authors listed have made a substantial, direct, and intellectual contribution to the work and approved it for publication.

## Conflict of Interest

The authors declare that the research was conducted in the absence of any commercial or financial relationships that could be construed as a potential conflict of interest.
